# Climatic forcing and individual heterogeneity in a resident mountain bird: legacy data reveal effects on reproductive strategies

**DOI:** 10.1098/rsos.221427

**Published:** 2023-05-24

**Authors:** Lasse Frost Eriksen, Thor Harald Ringsby, Hans Chr. Pedersen, Erlend B. Nilsen

**Affiliations:** ^1^ Centre for Biodiversity Dynamics (CBD), Department of Biology, Norwegian University of Science and Technology (NTNU), 7034 Trondheim, Norway; ^2^ Terrestrial Biodiversity Department, Norwegian Institute for Nature Research (NINA), 7034 Trondheim, Norway; ^3^ Faculty of Biosciences and Aquaculture (FBA), Nord University, 7713 Steinkjer, Norway

**Keywords:** trade-offs, state-dependent, clutch size, breeding time, repeatability

## Abstract

Optimization of clutch size and timing of reproduction have substantial effects on lifetime reproductive success in vertebrates, and both individual quality and environmental variation may impact life history strategies. We tested hypotheses related to maternal investment and timing of reproduction, using 17 years (1978–1994) of individual-based life history data on willow ptarmigan (*Lagopus l. lagopus*, *n* = 290 breeding females with *n* = 319 breeding attempts) in central Norway. We analysed whether climatic variation and individual state variables (age and body mass) affected the number of offspring and timing of reproduction, and individual repeatability in strategies. The results suggest that willow ptarmigan share a common optimal clutch size that is largely independent of measured individual states. While we found no clear direct weather effects on clutch size, higher spring temperatures advanced onset of breeding, and early breeding was followed by an increased number of offspring. Warmer springs were positively related to maternal mass, and mass interacted with clutch size in production of hatchlings. Finally, clutch size and timing of reproduction were highly repeatable within individuals, indicating that individual quality guided trade-offs in reproductive effort. Our results demonstrate how climatic forcing and individual heterogeneity in combination influenced life history traits in a resident montane keystone species.

## 1. Introduction

### 1.1. General introduction

Ongoing climate change will induce variation in individual life histories of vertebrate populations, which in turn will lead to perturbations in the population dynamics of single species that cascade through the foodweb [1]. Climate directly and indirectly affects abundance of food resources and therefore interferes with individuals' energy budgets and influences both acquisition and allocation of resources to growth, self-maintenance or reproduction [2]. For birds, maximizing fitness depends on an optimal investment in clutch size and timing of reproduction (e.g. [3,4]). Clutch size variation within a population is common, and climate-driven resource availability may affect the number of eggs laid [5]. In general, variation in number of offspring is often assumed to be closely linked to variation in food availability [6]. However, previous trade-off decisions [7,8] and individual state variables (e.g. age, body condition or social status) can also affect both ability to acquire resources and current allocation of resources [9,10]. As each individual is expected to optimize its own clutch size, a relationship between individual state variables and number of offspring has been predicted [10].

Reproductive output is generally expected to increase with age [11]. Central among the hypotheses explaining age-specific reproductive investment are the ‘constraint’ and ‘restraint’ hypotheses [10], and the ‘terminal investment’ hypothesis [12]. While the ‘constraint’ hypothesis posits that young birds are constrained in reproductive abilities directly or indirectly (e.g. through foraging abilities or subdominance in competition over territories), the ‘restraint’ hypothesis posits that young individuals are holding back on reproductive effort to allocate resources to survival or later reproduction efforts. The ‘terminal investment’ hypothesis predicts an end-of-life increase in reproductive effort, as there will be no need for resources for later reproductions [8,12,13]. Moreover, considerable research has investigated how parental nutritional state may affect reproductive success (e.g. [14]). Access to sufficient quality and quantity of nutrients is central to allocation of resources and may guide trade-offs affecting reproduction [11], including forcing individuals to invest more in survival and self-maintenance than in reproduction when resources are limited [15]. While individual optimization of clutch size indeed has been shown for some species (e.g. [16]), other studies suggest that an optimal clutch size (or litter size in mammals) can be independent of individual state if environmental conditions during the breeding and offspring-rearing period are unpredictable [17].

In addition to optimizing clutch size, fitness also depends on timing strategies. Adjustment of egg-laying date as a response to spring conditions is common in birds (e.g. [18–20]) and has been shown to be state-dependent for many species [21–24]. Such phenological adaptations are assumed to reflect trade-offs involving self-maintenance (fat storage and somatic growth) and reproduction, but also optimizing the number of offspring and ensuring enough time for offspring growth during summer [3]. There is ample evidence of a relationship between phenology and clutch size for many species, where early breeders generally produce more offspring [22,25–27]. However, early breeding may increase the risk of adverse weather extremes, with potential negative fitness consequences [15].

In this study, we test a set of hypotheses about maternal investment derived from several previous lines of research. We used a unique 17-year time series (1978–1994) of individual-based life history data on willow ptarmigan (*Lagopus l. lagopus*) from central Norway. High-latitude alpine systems like this are ideal for assessing trade-offs in reproductive investment, as the harsh environmental conditions induce strong selection pressure. Breeding seasons are relatively short [19], and hatching too early incurs high risks of sudden incidents of low temperatures or renewed snow cover, which can reduce offspring survival [28,29]. Following best practice procedures for confirmatory research [30], we pre-registered the background for the work and the hypotheses [31]. The specific hypotheses and the deduced predictions are described below.

### 1.2. Common versus state-dependent optimal clutch size

**Figure 1.**
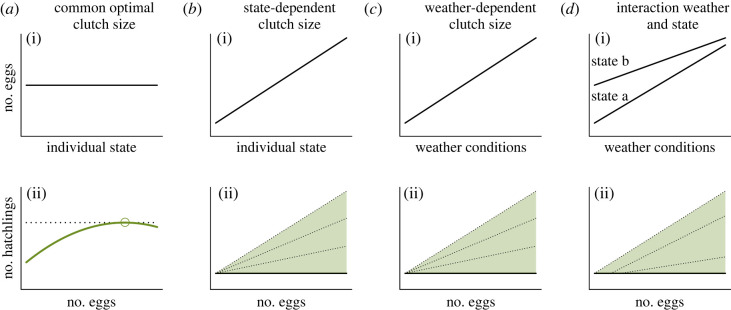
Conceptual representation of the two optimization modes contrasted, *common* (*a*) versus *state-dependent* (*b*) optimal clutch size (modified from [17]). The optimization models are extended with effects of weather (*c*) and interactions (*d*). With a common optimal clutch size, number of eggs is independent from individual state (*a*i) and number of hatchlings will be highest at the most common clutch size (*a*ii). In the case of state- or weather-dependent optimal clutch sizes (*b*i–*d*i), number of hatchlings will increase with clutch size (*b*ii–*d*ii). The filled triangles represent possible reproductive output, limited by the number of eggs laid, and dashed lines inside the triangles show examples of possible slopes depending on the composition of state and weather variables in the population.

We first conduct a conceptual quasi-replication [32] of a previous study, by testing the optimization model presented by Gaillard *et al*. [17] in a new taxon. In short, the model contrasts two competing hypotheses of clutch size optimization—*common optimal clutch size* versus *state-dependent clutch size*, respectively. As an extension to the original model (figure 1*a,b*), we expect an interaction between individual state and weather [33], predicting a stronger state dependence under harsh climatic conditions (figure 1*c*,*d*). Willow ptarmigan in Norway usually lay 8–12 eggs [34,35]. If optimal clutch sizes depend on individual state (figure 1*b*i) or weather conditions (figure 1*c*i), a change in a state or weather variable will change the number of eggs laid. By contrast, if clutch size is independent of measured state or weather variables, there will be no relationship between such variables and number of eggs, suggesting a common optimal clutch size across individuals (figure 1*a*i). We can expect either a positive or quadratic relationship between clutch size and number of hatchlings, and in case of a common optimal clutch size, reproductive output in terms of highest number of hatched chicks will peak at the most common clutch size observed (figure 1*a*ii). If, in contrast, the optimal clutch size is state- or weather-dependent, number of hatchlings will be positively (and linearly) correlated with observed clutch sizes, and number of hatchlings is determined by the composition of important state variables among the individuals, or weather conditions (figure 1*b*ii,*c*ii). Previous studies on subsamples of the data used here found no direct effects of maternal age on clutch size [36,37]. However, it is reasonable to hypothesize that maternal individuals that are young or low on fat-reserves may be constrained (*sensu* [10]) to a higher degree when environmental conditions are stressful [38], potentially affecting both clutch size and number of hatchlings, while older or larger individuals may be more capable to buffer against suboptimal conditions. Body mass has been shown to generally be a good indicator of body condition (i.e. fat content) in birds [39]. Based on the foundation outlined here, we predict that

(i) under the state-dependent model, low maternal body mass leads to reduced number of eggs laid
(ii) under the weather-dependent model, a decrease in local mean spring temperature or an increase in intensity of North Atlantic weather systems (indicated by an increased North Atlantic Oscillation (NAO) index value) is followed by a reduced number of eggs laid
(iii) young females (juveniles, less than 1 year) or females of low body mass are more sensitive to weather conditions than older (adults, greater than 1 year) or larger females, giving an interaction between weather and individual state on number of eggs laid
(iv) a decrease in local mean spring temperature or an increase in the NAO index is followed by a reduced number of hatchlings. This effect will be stronger for young females or females with low body mass

### 1.3. Onset of spring effects on timing and reproductive success

Onset of spring is expected to affect the timing of reproductive events, and general theory suggests that individuals with higher body mass [23,40] or higher age [38] may initiate egg-laying earlier. Young or low-weight females are expected to be more sensitive to climatic conditions than older or larger females (see also *predictions iii* and *iv*), due to a higher need for self-maintenance and accumulation of body fat before initiation of reproduction. Further, as indicated by Erikstad *et al*. [36] (based on the first 6 years of data) and a wide array of previous studies (e.g. [25,26,38,40,41]), early egg-laying is likely to be positively associated with reproductive output. Thus, we predict that

(v) early onset of spring resulting from higher spring temperatures or early snow-melt will be followed by early egg-laying
(vi) heavy females lay eggs earlier
(vii) adult females lay eggs earlier than juveniles
(viii) young or low-weight females have more delayed egg-laying under harsh climatic conditions than older or larger females
(ix) females that initiate egg-laying early lay more eggs
(x) females that initiate egg-laying early produce more hatchlings

Maternal nutrition is likely to affect reproduction for willow ptarmigan [42,43]. As body mass reserves used for reproduction to a large extent are accumulated from plants consumed in the weeks prior to reproduction [42,44], we predict that

(xi) females will be heavier in springs with high temperatures

### 1.4. Effects of individual quality beyond measured traits

The concept of ‘individual quality’ has seen different applications in the literature [45,46]. In the current study, we interpret quality as an unmeasured individual life history trait (or an abstract composite of several unmeasured traits), that may act on reproductive success directly or indirectly through other traits. Individual quality is assumed to influence variation in timing of breeding, which in turn is a major determinant of breeding success [23]. Thus, we expect that variation in reproductive success can be partially explained by individual characteristics beyond measured states and therefore predict that:

(xii) females repeat individual strategies with regard to timing of reproduction and number of offspring in consecutive breeding attempts

## 2. Materials and methods

### 2.1. Study system

Willow ptarmigan is a medium-sized (400–800 g depending on sex and season) resident tetraonid in Arctic and sub-/low-alpine tundra, with a circumpolar distribution [47,48]. They are relatively short-lived, with only a small proportion surviving to 4 years of age [38], although some individuals survive at least to the age of seven (unpubl. data from Norway; L.F.E. and E.B.N.). Females typically start breeding as yearlings [38], and the role of males in breeding after mating is mostly limited to predator defence [49]. Willow ptarmigan is a precocial species, but chicks are depending on their mother for thermoregulation and predator defence for several weeks after hatching [49,50]. The late winter diet is mainly based on twigs and buds from trees and shrubs, while the spring diet consists of field-layer plants where bilberry (*Vaccinium myrtillus)*, cottongrass (*Eriphorum* spp.), dwarf birch (*Betula nana*) and willows (*Salix* spp.) are central components [43]. The data for this study was collected in a sub-alpine to alpine area (900–1200 m.a.s.l.) of approximately 30 km^2^ on the eastern borders of Dovrefjell-Sunndalsfjella National Park in Norway (62°17'N, 09°36'E), in the years 1978–1994. The study area consists of two sub-areas that are divided by a paved road but otherwise connected and undistinguishable. See Pedersen *et al*. [51] for a detailed description of the study area.

### 2.2. Data collection

We used data from detailed monitoring of reproducing female willow ptarmigan during the breeding season (May–July) in the years 1978–1994. Although field effort was fairly equal between years, the number of females located varied, mainly caused by population fluctuations. We have no reason to believe that individuals nested prior to the start of monitoring. The females were found in mapped territories (cf. [51]) and by searching for clocker droppings (i.e. relatively large deposits of scat left by incubating hens when they occasionally leave the nest). The area was then searched using pointing dogs and beating the bushes with long sticks, trying to flush the incubating female. When nests were detected, females were captured on the nest using throw-nets. Birds were weighed to the nearest 5 g, and age group (juvenile, less than 1 year; adult, greater than 1 year) was determined based on pigmentation of the 8th and 9th primaries [52]. All birds were ringed with metal leg rings and some individuals were instrumented with a radio transmitter (not part of the present study). Initiation of incubation for each female was estimated by performing flotation tests on eggs [53] and clutch initiation dates were estimated by assuming that after the first egg was laid the female laid one egg per day continuously [54]. As willow ptarmigan incubate for approximately 21 days after laying the last egg [40,55], monitoring of the nest until expected hatching further improved the estimation of oviposition and incubation start dates. Number of eggs in the nest was counted at the time of capturing the female. Number of hatched chicks leaving the nest was estimated as number of eggs, minus number of unhatched eggs and dead hatched chicks found in the nest. Predation events could be separated from hatching by inspecting the eggs, as predators either damaged or removed the eggs while pipped eggshells signified hatched chicks. Similar to Kvasnes *et al*. [56], we defined different time periods during spring-summer where weather is expected to be important for reproductive success. To investigate detailed mechanisms, we defined *individual-based* periods according to the specific dates for each single nest, including a pre-oviposition, a pre-incubating and an incubating period. Following the length of the incubating period [55], all three periods were set as 21-day intervals, and the periods were not used simultaneously in any analyses. In addition to the individual-based weather periods, we used seasonal time-windows of spring temperature and snow depth, based on the median laying date of first egg for initial clutches over all individuals and years, to reveal *general* effects of onset of spring. See table 1 for an overview of all weather parameters used. All weather data are publicly available through The Norwegian Meteorological Institute (https://www.met.no/en/free-meteorological-data/Download-services). We used temperature and snow depth data recorded at Fokstua Meteorological Station, *ca* 30 km south of the study area and within the same mountain region. As it is unclear which climatic variables would be most appropriate and the breeding period stretches over May, June and July, we also used the seasonal station-based NAO index for May–July as an alternative long-term climate variable. The NAO indexes are produced by NCAR's Climate Analysis Section based on Hurrell [57] and are accessible from Climate Data Guide (https://climatedataguide.ucar.edu/climate-data/hurrell-north-atlantic-oscillation-nao-index-station-based).

**Table 1.** Weather parameters used to analyse the relationships between climatic forcing, individual characteristics and reproductive success. ‘ind/gen’ indicate if the weather parameter is calculated according to the breeding dates for each individual bird (ind), or if it is based on a common general time period (gen) with the median laying date of first egg for initial clutches over all individuals and years (i.e. 27 May) as end-date. All temperature (°Celsius) and snow depth (mm) parameters are arithmetic means of the daily means over the time-window.

abbr.	parameter	ind/gen	time period
Temp_pre-ovi_	pre-oviposition temp.	ind	1–21 days prior to first egg laid
Temp_pre-inc_	pre-incubation temp.	ind	1–21 days prior to incubation start
Temp_inc_	incubation temp.	ind	21 days of incubation
NAO_May–July_	North Atlantic Oscillation	gen	seasonal NAO for May–July
Snow_spring_	spring snow depth	gen	15, 30, 45 and 60 days prior to median laying date
Temp_spring_	spring temp.	gen	15, 30, 45 and 60 days prior to median laying date
Temp_multiple_	spring temp. (multiple)	gen	exploratory approach including all periods in tens 1–60 days prior to median laying date (e.g. 1–10, 1–20, 11–20 days etc.)

### 2.3. Statistical analyses

The data comprised a total of 319 breeding willow ptarmigan females, including 29 females that were monitored in more than one breeding season. Not all data was collected for all individuals or nesting seasons, and observations with missing data in any parameter used for a specific analysis were removed before conducting the analysis. Willow ptarmigan have only one brood per year but may renest if the first nesting attempt is unsuccessful [34,58]. Based on previous research [36,58], we assumed that nests initiated 16 days or more after the first nest of the season was initiated, was a renesting attempt. We used only initial nesting attempts in our analyses, because renests are based on a different resource base and environmental conditions. One observation with 16 eggs (in 1991) in the nest was removed from clutch size analyses as an outlier, as the second largest clutch in the entire dataset was 13 eggs. Correlated variables (age group and weight, several climatic variables and individual timing events) were not included in the same model. To account for weight loss during incubation, we used residual weight from the relationship ‘*weight∼incubation time at capture’* as a predictor. *Year effects* were included as random intercepts in mixed-effects models (see details for each tested hypothesis below) for optimal clutch size and timing of reproduction, in order to estimate the residual variation caused by correlations within a year (e.g. caused by annual variation in predation pressure or unmodelled climatic effects). We did not include mother ID as a random effect in our main analyses due to few repeated measurements, but we present alternative models with mother ID as a random effect in the last section of electronic supplementary material, appendix A, with very similar results and the same main conclusions. To avoid overfitting, we included only one interaction effect in any model. Continuous predictor variables were standardized before analyses (i.e. scaled and centred by extracting the mean and dividing on the standard deviation) to facilitate comparisons. All modelling was performed with the statistical software R (version 4.2.2; [59]). Model selection was based on sample-size corrected Akaike information criterion (AIC_c_) [60], and we considered models with ΔAIC_c_ < 2 as competing models. Model fit was assessed by inspection of residuals versus fitted values and distribution of random effect intercepts when applicable. See electronic supplementary material, appendix A for expanded general descriptives of the data, R code (with data management code prior to analyses in electronic supplementary material, appendix B), and a step-by-step walkthrough for each analysis.

#### 2.3.1. Testing the optimal clutch size hypotheses (predictions i–iv)

We first analysed the variation in number of eggs in relation to the state variables *age group* and *body mass* and the climatic variables *Temp_pre-inc_* and *NAO_May–July_* (*model 1, predictions i–iii*, *n* = 154, figure 1*a*i–*d*i). In the second part of the optimal clutch model, we analysed the variation in number of chicks that successfully hatched, dependent on number of eggs produced (including quadratic effects of number of eggs to reveal any optimal clutch size), the state variables and the climatic variables *Temp_inc_* and *NAO_May–July_* (*model 2, prediction iv*, *n* = 87, figure 1*a*ii–*d*ii). We included interactions between egg number, state variables and climatic variables, but not interactions with quadratic effects of egg number as this would give added complexity and not directly reflect the hypotheses being tested. Modelling nest success as a binary response indicated no evidence of a relationship between clutch size and nest failure, thus, we analysed number of hatchlings without considering nests that failed completely. Nests or females that had been subjected to experimental manipulation between egg-laying and hatching (not part of the present study, see [61]) were excluded from the analyses. When assessing variation in number of hatchlings, we created a cut-off excluding nests where more than one-third of the eggs did not hatch, as these observations (9.4% of the sample) with excessive reduction in clutch size between eggs laid and chicks hatched was most likely caused by other mechanisms (partial predation, eggs kicked out by the hen or a high number of unhatched/unfertilized eggs) than what we address in this study. The different causes of excessive reduction in clutch sizes were relatively rare events. Accordingly, including these nests would certainly contribute to conceal the ecological mechanisms we address in the present study. Furthermore, they strongly interfered with model convergence and model validation due to excessive variation, thus, we excluded these nests from the hatchling analyses. Dispersion tests using the *DHARMa* package (version 0.4.6; [62]) revealed that our data was underdispersed (both before and after exclusion of nests with excessive variation in the hatchling analyses), meaning that Poisson model assumptions were violated. Consequently, we opted for modelling reproductive output by use of Conway–Maxwell Poisson distribution (a generalization of the Poisson family allowing for both underdispersion and use of random effects) in generalized linear mixed models (GLMMs) with the R package *glmmTMB* (version 1.1.5; [63]).

#### 2.3.2. Testing the optimal timing hypotheses (predictions v–xi)

In order to explain the variation in timing of egg-laying (*model 3, predictions v–viii*, *n* = 155), we modelled the effects of individual state (*age group* and *body mass*), onset of spring (*Snow*_spring_ and *Temp*_spring_) and additional climatic variables (*NAO*_May–July_ and *Temp*_pre-ovi_) with Gaussian error distribution in linear mixed models (LMM), using the package *lme4* (version 1.1–31; [64]). To remove the temporal effects on pre-oviposition temperature, we used the residuals from the relationship ‘*Temp_pre-ovi_∼timing of first egg laid + random intercept of year’* as a predictor instead of raw values of pre-oviposition temperature. We further tested if variation in timing strategy affected the number of eggs (*model 4, prediction ix*, *n* = 182) or hatchlings (*model 5, prediction x*, *n* = 108) by the use of GLMMs with Conway–Maxwell Poisson distribution (see above), where individual *time of first egg laid* and *time of incubation start*, as well as their quadratic effects for assessing any optimal timing, were used as fixed-effect variables. When modelling number of hatchlings (*model 5*), we used the same cut-off for excluding nests with excessive reduction in clutch size between eggs laid and chicks hatched as previously described (7.7% of the sample). In *model 6* (*prediction xi, n* = 164), we tested the relationship between pre-laying temperatures and body mass using LMMs, including the *number of days incubated* at capture time as a fixed effect to account for weight loss during incubation. With little prior knowledge of which part of the pre-laying period would be most important, we chose an exploratory selection of climatic windows before the median laying date (i.e. 27 May) for initial clutches in our dataset (*Temp_multiple_*, cf. Table 1).

#### 2.3.3. Testing the individual quality hypothesis (prediction xii)

To test our third hypothesis, we modelled the consistency within individuals by estimating repeatability in *time of first egg laid* (*model 7*, total *n* = 172 where 31 were repeated observations from 15 resampled individuals) and *clutch size* (*model 8*, total *n* = 224, 33 rep. obs. from 16 ind.), for individual females followed over more than 1 year (range 2–3 years) (*prediction xii*). We estimated the adjusted repeatabilities (*sensu* [65]), fitting LMMs with the package *rptR* (version 0.9.22; [66]) while controlling for fixed-effect covariates found to be important in the previous analyses (i.e. covariates from *model 3* for estimating repeatability in timing and *model 4* for repeatability in clutch sizes). Although the clutch size model (*model 8*) is based on count data, which is usually modelled with GLMM's, the data is underdispersed (see above). The package *rptR* is not recommended for modelling underdispersed count data with Poisson distribution [66], but as the clutch size repeatability model (*model 8*) had normally distributed residuals and passed model validation, fitting also this model as an LMM with Gaussian error distribution should be appropriate.

## 3. Results

### 3.1. Optimal clutch size

The most commonly observed clutch sizes for initial clutches were 9 or 10 eggs, together accounting for 51.6% of all initial clutches. As expected, initial clutches consistently had a higher number of eggs than renestings (electronic supplementary material, appendix A). Based on model selection guided by AIC_c_, we did not find any clear evidence that initial clutch size varied as a function of maternal state (*prediction i* and *iii*) or included weather parameters (*prediction ii* and *iii*) (*model 1*; table 2; full model selection tables are shown in electronic supplementary material, appendix A). Although maternal weight, NAO_May–July_ and pre-incubating temperature were present among the top models, the biological effects were small and there was high parameter uncertainty in all these models. This indicates a common optimal clutch size across individuals, with limited support to *predictions i–iii*. The mean clutch size for all females in all years was 9.7 (95% confidence interval (CI) [9.25, 10.15], range 5–13 eggs).

**Table 2.** Optimal clutch size model selection tables based on AIC_c_. Top models with *Δ*AIC_c_ < 2 and null model (intercept only) for each analysis are shown in ranked order. The parameter ‘weight’ refers to maternal weight at capture. See electronic supplementary material, appendix A for full model selection tables and parameter estimates and confidence intervals (CI) for all competing top models.

model	*Δ*AIC_c_	*Δ*logLik	weight
*1) optimal clutch size: number of eggs*			
null model	0.0	0.0	0.22
weight	0.3	0.9	0.19
NAO_May–July_	1.8	0.2	0.09
Temp_pre-inc_	1.9	0.1	0.09
*2) optimal clutch size: number of hatchlings*			
eggs + eggs^2^	0.0	54.2	0.12
eggs + eggs^2^ + weight + eggs × weight	0.2	56.5	0.11
eggs + eggs^2^ + NAO_May–July_	0.8	55.0	0.08
eggs + eggs^2^ + weight + eggs × weight + NAO_May–July_	1.0	57.3	0.07
eggs + eggs^2^ + weight	1.8	54.5	0.05
null model	104.0	0.0	0.00

**Figure 2.**
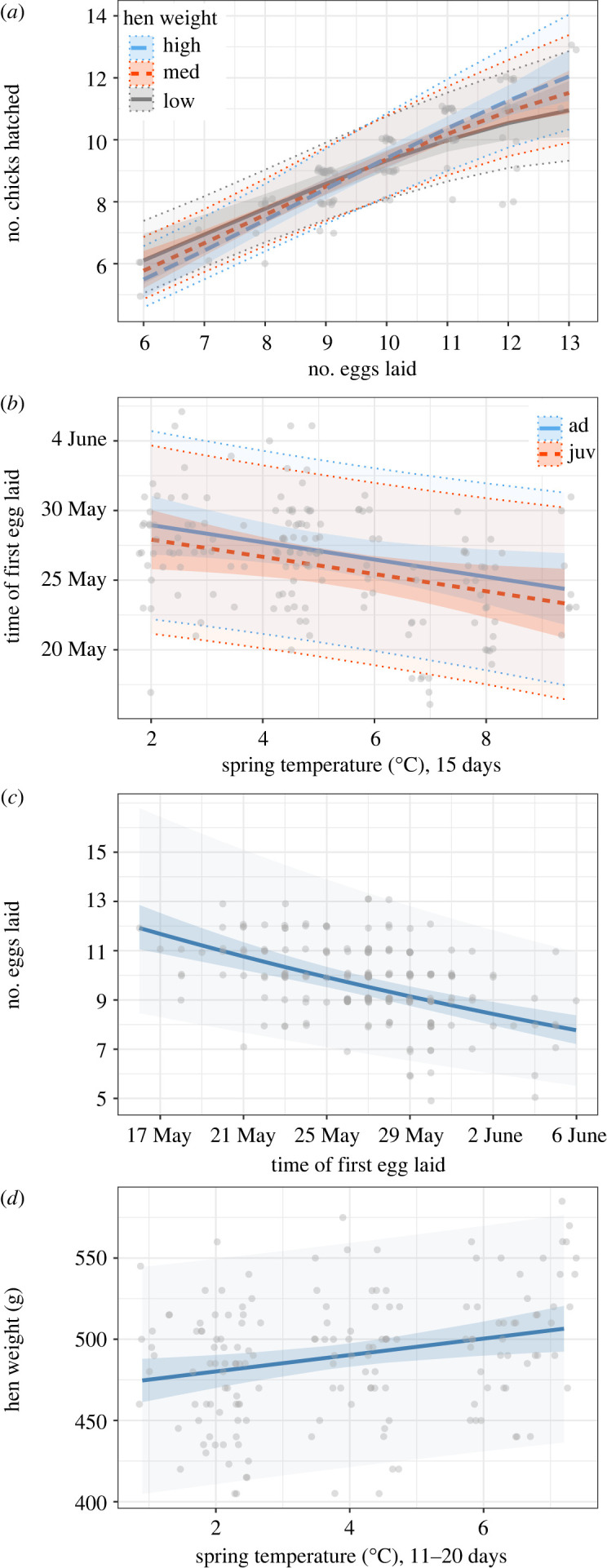
The effects of (*a*) clutch size and maternal weight on number of willow ptarmigan chicks hatched (showing the 10th, 50th and 90th percentile of weights), (*b*) age group and spring temperature (over 15 days) on timing of egg-laying, (*c*) timing of breeding on clutch size and (*d*) spring temperature (over day 11–20 before egg-laying) on body mass of incubating females. Densely coloured ribbons are 95% confidence intervals for the population level effects of fixed terms, at the mean values of year as random term. Expanded lighter ribbons are prediction intervals including random year effects. Raw data are shown with jittered points.

For our analysis of number of hatchlings under the optimal clutch size hypothesis (*model 2*; table 2), we base our inference on the two highest ranking models. Both these models indicated a common optimal clutch size across individuals, where the number of chicks produced increased with increasing clutch size until reaching a peak, and where the model predicted declining hatching success at clutch sizes higher than the optimum. However, while the relative gain of additional eggs decreased at larger clutch sizes, the data reveal that clutch sizes were still limited to a level well below the potential common optimum (figure 2*a*, where the model prediction peak is not shown as it is outside of the area where data are present). We found no support for *prediction iv* (number of hatchlings affected by an interaction between weather and maternal state). However, in the second-ranked model (table 2), there was an interaction effect between female body mass and the number of eggs laid on the number of hatchlings (*β*_eggs_ = 0.145, CI [0.124, 0.166]; *β*_eggs_^2^ = −0.019, CI [−0.035, −0.003]; *β*_weight_ = 0.003, CI [−0.017, 0.023]; *β*_eggs × weight_ = 0.019, CI [0.001, 0.037]), where hens with high weight produced more hatchlings from large clutches than lighter hens did.

### 3.2. Optimal timing

There was ample variation in timing of breeding among individual willow ptarmigan females over the years, with earliest start of initiating egg-laying at 16 May and latest (initial clutch) at 6 June. All top-ranking models suggest a weather-dependent optimal timing (*model 3*; table 3), giving strong support for our *prediction v* (i.e. higher spring temperature was followed by early egg-laying). Models including time periods of 15, 30 and 60 days prior to egg-laying all have merit, where e.g. a mean increase of 1°C over 15 days led to a 0.62 days (CI [−1.136, −0.097]) advance in egg-laying date (figure 2*b*). We found no effects of maternal weight on timing, giving no support for *prediction vi*. Surprisingly, several models showed a clear tendency of an opposite age-dependent effect of what we anticipated in *prediction vii*, where juveniles started egg-laying earlier than adults (*β*_age.juv_ = −1.04, CI [−2.152, 0.086). There were indications of an interaction effect between age group and spring temperature, but notable parameter uncertainties imply no or weak support to *prediction viii* (timing affected by an interaction between weather and maternal state).

**Table 3.** Optimal timing model selection tables based on AIC_c_. Top models with *Δ*AIC_c_ < 2 and null model for each analysis are shown in ranked order. ‘Temp_x_ (days)’ indicates the period that mean temperatures are calculated over. See electronic supplementary material, appendix A for full model selection tables and parameter estimates and confidence intervals (CI) for all competing top models.

model	*Δ*AIC_c_	*Δ*logLik	weight
*3) optimal timing: day of first egg laid*			
age group + Temp_spring_ (15 days)	0.0	4.4	0.14
age group × Temp_spring_ (15 days)	0.9	5.0	0.09
age group × Temp_spring_ (30 days)	1.0	5.0	0.08
age group + Temp_spring_ (30 days)	1.1	3.9	0.08
Temp_spring_ (15 days)	1.1	2.8	0.08
age group + Temp_spring_ (60 days)	1.4	3.7	0.07
null model	4.6	0.0	0.01
*4) optimal timing: number of eggs*			
day of first egg laid	0.0	20.2	0.67
day of first egg laid + day of first egg laid^2^	1.4	20.5	0.33
null model	38.3	0.0	0.00
*5) optimal timing: number of hatchlings*			
day of first egg laid + day of first egg laid^2^	0.0	11.6	0.55
day of first egg laid	0.4	10.3	0.45
null model	18.8	0.0	0.00
*6) optimal timing: body mass*			
days incubated + Temp_multiple_ (11–20 days)	0.0	3.1	0.30
null model	4.1	0.0	0.04

In strong support of our *prediction ix*, we found clear evidence of a relationship between timing of breeding and clutch size. The number of eggs laid was best explained by a negative linear relationship with day of initiating egg-laying (*model 4*; table 3; figure 2*c*). The top model indicated a mean reduction of 0.20 eggs for each day egg-laying was delayed (*β*_day of first egg laid_ = −0.083, CI [−0.107, −0.058]). Further, we found a clear relationship between timing of breeding and the number of hatched chicks, giving an equally strong support for *prediction x* (*model 5*; table 3). The simpler model with linear effects of day of first egg laid (*β*_day of first egg laid_ = −0.083, CI [−0.119, −0.047]) indicated a reduction of 0.18 hatchlings per day egg-laying was delayed. Here, also a quadratic relationship with higher parameter uncertainty was supported (*β*_day of first egg laid_ = −0.084, CI [−0.119, −0.050]; *β*_day of first egg laid_^2^ = −0.019, CI [−0.041, 0.004]). An F-test showed no evidence of a difference between the variance in first egg laid and last egg laid (F_107_ = 1.274, *p* = 0.213).

There was strong support for our *prediction xi* of a positive relationship between pre-laying temperatures and maternal body mass. The variation in body mass was best explained (*model 6*; table 3) by Temp_multiple_ (11–20 days before the median laying date 27 May), i.e. a 10 day window in the first half of May in our study area and time period, indicating that a 1°C change in temperature was followed by a 5.1 g increase in body mass (figure 2*d*; *β*_incubation time_ = −9.067, CI [−14.601, −3.406], *β*_temp.11–20_ = 9.773, CI [2.524, 16.759]).

### 3.3. Individual quality

In full support of *prediction xii*, our results show a high level of repeated strategies among the individuals observed over more than one breeding season. Adjusted repeatability (R), controlled for the effects of age group and mean temperature (0–60 days), was fairly high for timing of first egg laid (*model 7*; *R* = 0.59, CI [0.31, 0.86]). For clutch sizes, controlled for timing of first egg laid, the level of repeatability was even higher (*model 8*; *R* = 0.74, CI [0.56, 0.89]).

## 4. Discussion

In the present study, we have demonstrated important relationships between key life history traits and local environmental conditions in a long-term study of a resident low-alpine bird. Using willow ptarmigan as a model species, we tested models of reproductive strategies in relation to individual heterogeneity and climatic variation in a harsh mountanous environment. In our initial analyses, we found no direct effects of maternal state or climatic conditions on clutch sizes. However, when disentangling the different components of reproduction, we found clear evidence that elevated spring temperatures advanced breeding, and strong support for our predictions of increased reproductive output with early breeding. Although the weather effect on timing did not directly influence clutch sizes with our chosen climatic parameters, we see a clear indirect path where temperature modulates breeding time and, consequently, affects reproductive output. Further, we found a positive effect of spring temperature on female body mass, but no direct effect of female mass on breeding time or clutch size, although body mass interacted with clutch size regarding the ability to produce hatchlings. Although Labocha & Hayes [39] found that body mass was an equally good descriptor of body condition (i.e. fat content) as any other index, it is likely to be confounded with structural size to some degree [39], possibly contributing to masking any direct effects of female mass in our results. Our prediction of individual quality was fully supported, with clear evidence of repeated timing- and clutch size-strategies among recaptured individuals.

In the first part of our study, we conducted a quasi-replication of previous work, by testing competing models of optimal clutch size [17] for our model species. The highest ranking model in our model selection procedure was the null model. Thus, number of eggs laid was largely independent of both the included climatic factors (as found for *Lagopus l. scotica* [41], but see Steen *et al*. [33]) and individual state, giving little support to *predictions i–iii*. Although a model including maternal weight was ranked second, parameter uncertainty was substantial. Given the connection between temperature, breeding time and clutch size, it is possible that a wide selection of general weather parameters in our optimal clutch analysis could have returned an apparent direct link between weather and clutch size. However, our focus here was to test hypotheses of detailed mechanisms, thus, we chose to keep our *a priori* set weather parameters.

The lack of clear effects of maternal states on optimal clutch size is in agreement with one previous study on *Lagopus* spp*.* (*L. l. alexandrae*; [67]) but in contrast with the age effects (*L. l. lagopus, L. leucura*; [38]) and weight effects (*L. l. lagopus*; [35], *L. muta hyperborea*; [68]) found by others. The state- and weather-independent clutch sizes in our study may indicate limited physiological costs of egg production, informing a long-standing debate on the matter [69]. It also leads to the assumption of a common optimal clutch size across individuals, in line with the findings for Eurasian lynx (*Lynx lynx*; [17]). As argued by Gaillard *et al*. [17], number of offspring may be independent of maternal state if environmental conditions after breeding are unpredictable, which is the case also for low-alpine willow ptarmigan. However, while the marginal effect of laying one additional egg was reduced at higher clutch sizes, clutch sizes were still confined well below the optimum, i.e. the level yielding the highest output in terms of hatched chicks. This may imply that clutch sizes are limited due to *a)* a time-limitation trade-off to allow sufficient time for chick growth before winter, *b)* territory quality affecting available resources, *c)* a limit on the number of young the parents are able to raise through the summer or *d)* trade-off decisions affecting allocation of available resources to reproduction versus short-term or long-term survival. As willow ptarmigans are able to renest if the initial clutch is lost [58], allowing sufficient time for chick growth is less likely to be the only explanation. Although territory quality has been found to be important for other species (e.g. [70]), Steen *et al*. [71] found no link between territory and food quality in our study population. Regarding a maximum number of young that can be raised, experiments with brood-enlargement show that in most bird species studied, parents were able to raise more young than they had eggs [72]. This is likely to be correct also for willow ptarmigan, being a precocial species, but presumably it is also weather-dependent; although the chicks are self-provided with food, they are dependent on their mother to warm up between feeding sessions [50,73]. Thus, given the unpredictable climatic summer conditions associated with the low-alpine habitat, an excessive number of chicks may increase competition among brood-mates and lead to reduced warming opportunities for the entire brood. Consequently, a bet-hedging strategy [74], i.e. limiting the investment and laying a clutch size below the optimum, may potentially yield the highest reproductive output (e.g. [74]). This strategy is also more resource-conserving than maximizing the clutch size, with potential benefits for long-term reproduction and survival [2]. We do not have data to investigate potential trade-offs between clutch size and future survival or fecundity for the individual chick, but it should be noted that natal brood size could in itself affect individual life histories [75] and, thus, optimal clutch sizes. For example, Spagopoulou *et al*. [75] experimentally demonstrated that collared flycatcher *(Ficedula albicollis*) females from smaller broods had higher reproduction early in life, but faster senescence and higher late-life mortality, than females raised in larger broods.

The analysis of number of hatchlings indicated a common optimal clutch size with interactive effects between number of eggs and maternal body mass. However, we did not find support for the prediction that individual state interacted with weather conditions. It appeared that light-weight females had a lower optimum than heavier females, i.e. the light-weight individuals were less capable of producing a high number of hatchlings from a large clutch size. This indicates that even if light-weight individuals may allocate a relatively higher amount of resources to egg production, the hatching success is reduced compared to heavier females, presumably due to a higher need for foraging during incubation [76].

We found evidence of phenotypic plasticity as female willow ptarmigan adjusted their timing of egg-laying to the onset of spring, supporting *prediction v*. This is in line with the study of Fletcher *et al*. [41] on red grouse (*L. l. scotica*), as well as research on a number of other avian species (e.g. [77]). Plasticity in timing should reflect an ability for the female to accumulate the necessary resources from fresh nutrient-rich plant forage [3,42]. Our results indicate that the weight-increase is most dependent on temperatures in early spring (11–20 days before egg-laying, supporting *prediction xi*), but as female grouse begin gaining weight about a month before egg-laying [42], we can assume that several environmental factors work in concert. Although the ability to adjust timing is state-dependent for many species [22,38,40], we did not see an advancement of breeding due to higher age or body mass (*predictions vi–viii*). Instead, our analyses revealed that juveniles were more than a day *earlier* than adults. That inexperienced females start earlier is particularly interesting as early breeding is followed by a higher number of offspring (supporting our *predictions ix and x*, see also [38,40,41]). We suspect that this is caused by adults showing restraint (*sensu* [10]), possibly based on previous experience of stochastic weather events in the early post-hatching period when harsh weather can severely affect food intake of offspring [29,73]. If this is the case, we see a state-dependent adjustment of timing working in the opposite direction than expected, where the delayed timing may have constituted an adaptation to an increased risk of ‘extreme’ weather [15].

Individual quality must be assumed to have affected the observed high levels of repeatability in breeding strategies. We found clear evidence for individual consistency in clutch sizes and breeding time, in strong support of *prediction xii*. Our results show a much higher clutch size repeatability (*R* = 0.74) than Myrberget [78] found in a willow ptarmigan population *ca* 850 km northeast from our study area (*R* = 0.23). Also the high repeatability we found in breeding time (*R* = 0.59) is in sharp contrast with e.g. the moderate repeatability in coot (*Fulica atra*) breeding time (*R* = 0.32) found by Perdeck & Cavé [21]. The simultaneous lack of age constraints in our results indicates that the composition of phenotypes in the sample population was probably more important than age distribution for breeding time and clutch sizes, and that individual traits and climatic variation together had great impact on reproductive success.

Our findings suggest that a common optimal clutch/litter size, largely independent of maternal states, may be a general feature across many species breeding and raising offspring in unpredictable environmental conditions. Furthermore, it can be expected that fluctuating climatic conditions in this high-latitude alpine system will result in a fluctuating selection on timing of reproduction and maternal weight, and consequently on clutch sizes. However, an increased risk of stochastic weather events during brooding and chick-rearing as a result of earlier breeding may have adverse effects on the number of juveniles that survive through their first summer [79]. With individual heterogeneity affecting trade-offs involving life history traits such as reproductive rates, this can have direct effects on population dynamics [80]. Accordingly, individual capacity to adapt through plasticity and eco-evolutionary processes is, thus, crucial in the face of climatic changes. We expect this to be a general feature for many species breeding in unpredictable high-latitude mountainous environments, where phenotype composition in meta-populations may be of high importance for the ability to adapt.

## Data Availability

Data used for this article is available from Dryad (https://doi.org/doi:10.5061/dryad.tdz08kq3z) [81]. Weather data are available from https://www.met.no/en/free-meteorological-data/Download-services. NAO data are available from https://climatedataguide.ucar.edu/climate-data/hurrell-north-atlantic-oscillation-nao-index-station-based. Supplementary material is available online [82].
